# Foot Plantar Pressure Measurement System: A Review

**DOI:** 10.3390/s120709884

**Published:** 2012-07-23

**Authors:** Abdul Hadi Abdul Razak, Aladin Zayegh, Rezaul K. Begg, Yufridin Wahab

**Affiliations:** 1 School of Engineering and Science, Victoria University, Melbourne, VIC 3032, Australia; E-Mail: Aladin.Zayegh@vu.edu.au; 2 Faculty of Electrical Engineering, Universiti Teknologi MARA, Shah Alam 40000, Malaysia; 3 School of Sport and Exercise Science (SES) and Institute of Sport, Exercise and Active Living (ISEAL), Victoria University, Melbourne, VIC 3032, Australia; E-Mail: Rezaul.Begg@vu.edu.au; 4 Centre for Industrial Collaboration, School of Microelectronic Engineering, Universiti Malaysia Perlis, Arau 02600, Malaysia; E-Mail: yufridin@unimap.edu.my

**Keywords:** foot plantar pressure, pressure sensor, wireless system

## Abstract

Foot plantar pressure is the pressure field that acts between the foot and the support surface during everyday locomotor activities. Information derived from such pressure measures is important in gait and posture research for diagnosing lower limb problems, footwear design, sport biomechanics, injury prevention and other applications. This paper reviews foot plantar sensors characteristics as reported in the literature in addition to foot plantar pressure measurement systems applied to a variety of research problems. Strengths and limitations of current systems are discussed and a wireless foot plantar pressure system is proposed suitable for measuring high pressure distributions under the foot with high accuracy and reliability. The novel system is based on highly linear pressure sensors with no hysteresis.

## 1. Introduction

The development of miniature, lightweight, and energy efficient circuit solutions for healthcare sensor applications is an increasingly important research focus given the rapid technological advances in healthcare monitoring equipment, microfabrication processes and wireless communication. One area that has attracted considerable attention by researchers in biomedical and sport related applications is the analysis of foot plantar pressure distributions to reveal the interface pressure between the foot plantar surface and the shoe sole. Typical applications are footwear design [1], sports performance analysis and injury prevention [2], improvement in balance control [3], and diagnosing disease [4]. More recently innovative applications have also been made to human identification [5], biometric [6], monitoring posture allocation [7] and rehabilitation support systems [8–10]. Based on this research it is clear that techniques capable of accurately and efficiently measuring foot pressure are crucial to further developments.

The plantar pressure systems available on the market or in research laboratories vary in sensor configuration to meet different application requirements. Typically the configuration is one of three types: pressure distribution platforms, imaging technologies with sophisticated image processing software and in-shoe systems. In designing plantar pressure measurement devices the key requirements are spatial resolution, sampling frequency, accuracy, sensitivity and calibration [11]. These requirements will be discussed in detail later.

In-shoe foot plantar sensors have paved the way to better efficiency, flexibility, mobility and reduced cost measurement systems. For the system to be mobile and wearable for monitoring activities of daily life, the system should be wireless with low power consumption. Wireless in-shoe foot plantar measurement systems have potential application to data transfer communication systems, miniaturized biomedical sensors and other uses. For compact, low cost devices for short-range wireless applications an on-chip antenna is a practical solution. On-chip antenna implementation is feasible with the assistance of rapid scaling of low cost complementary metal-oxide-semiconductor (CMOS) technology. The feasibility of creating circuits and systems to operate at lower frequency bands and subsequently reducing the antenna size using on-chip antennas has been discussed [12,13].

This review will first summarize the existing methods for measuring foot plantar pressure and the advantages and disadvantages of a range of commercial pressure sensors used in published research. Subsequently, the discussion will introduce a micro-electromechanical (MEMS) pressure sensor that has considerably enhanced performance characteristics. Finally various solutions presented by researchers to measure foot plantar pressure using in-shoe system will be reviewed. The review critically examines the devices used in measuring systems, such as sensors, processing units and wireless transmitters. The paper compares the compactness, power consumption, number of sensors and placements of sensors used in published systems and we propose a new system, the MEMS sensor. The MEMS sensor will interface with a wireless data acquisition (DAQ) unit, which is a full-custom design using CMOS technology. This novel solution will be on a single chip making it highly compact and low in power consumption.

The paper is divided into eight sections. Section 2 presents the requirements for plantar pressure measurement systems. The foot plantar pressure measurement environment will be discussed in Section 3. Section 4 will describe the application requirements of foot plantar sensors. Section 5 documents the commercial foot plantar pressure measurement sensors in detail. The wireless foot plantar pressure systems will be reviewed in Section 6. Section 7 presents our proposed new approach to recording foot plantar pressure and the system's design. Finally, Section 8 discusses the suitability of the proposed system and conclusions.

## 2. Needs for Plantar Pressure Measurement

Feet provide the primary surface of interaction with the environment during locomotion. Thus, it is important to diagnose foot problems at an early stage for injury prevention, risk management and general wellbeing. One approach to measuring foot health, widely used in various applications, is examining foot plantar pressure characteristics. It is, therefore, important that accurate and reliable foot plantar pressure measurement systems are developed. One of the earliest applications of plantar pressure was the evaluation of footwear. Lavery *et al.* [14] in 1997 determined the effectiveness of therapeutic and athletic shoes with and without viscoelastic insoles using the mean peak plantar pressure as the evaluation parameter. Since then there have been many other studies of foot pressure measurement; for example, Mueller [15] applied plantar pressure to the design of footwear for people without impairments (*i.e.*, the general public). Furthermore, Praet and Louwerens [16] and Queen *et al.* [17] found that the most effective method for reducing the pressure beneath a neuropathic forefoot is using rocker bottom shoes and claimed the rocker would decrease pressure under the first and fifth ray (metatarsal head). The metatarsal heads are often the site of ulceration in patients with cavovarus deformity. Queen *et al.* indicated that future shoe design for the prevention of metatarsal stress fractures should be gender specific due to differences in plantar loading between men and women.

With regard to applications involving disease diagnosis, many researchers have focused on foot ulceration problems due to diabetes that can result in excessive foot plantar pressures in specific areas under the foot. It is estimated that diabetes mellitus accounts for over $1 billion per year in medical expenses in the United States alone [18]. Diabetes is now considered an epidemic and, according to some reports, the number of affected patients is expected to increase from 171 million in 2000 to 366 million in 2030 [19]. Improvement in balance is considered important both in sports and biomedical applications. Notable applications in sport are soccer balance training [20] and forefoot loading during running [21]. With respect to healthcare, pressure distributions can be related to gait instability in the elderly and other balance impaired individuals and foot plantar pressure information can be used for improving balance in the elderly [22]. Based on the above discussion, it is crucial to devise techniques capable of accurately and efficiently measuring foot pressure.

## 3. Foot Plantar Pressure Measurement Environments

There are a variety of plantar pressure measurement systems but in general they can be classified into one of two types: platform systems and in-shoe systems.

### 3.1. Platform Systems

**Figure 1.**
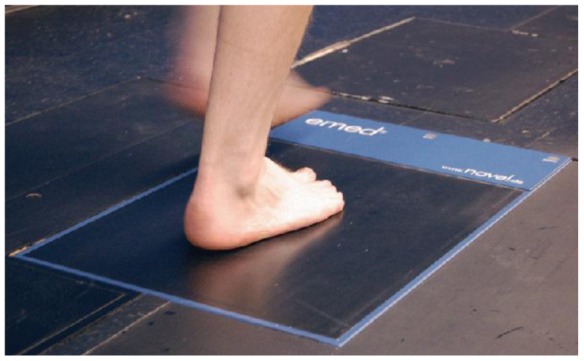
A platform-based foot plantar pressure sensor emed^®^ by Novel [24].

**Figure 2.**
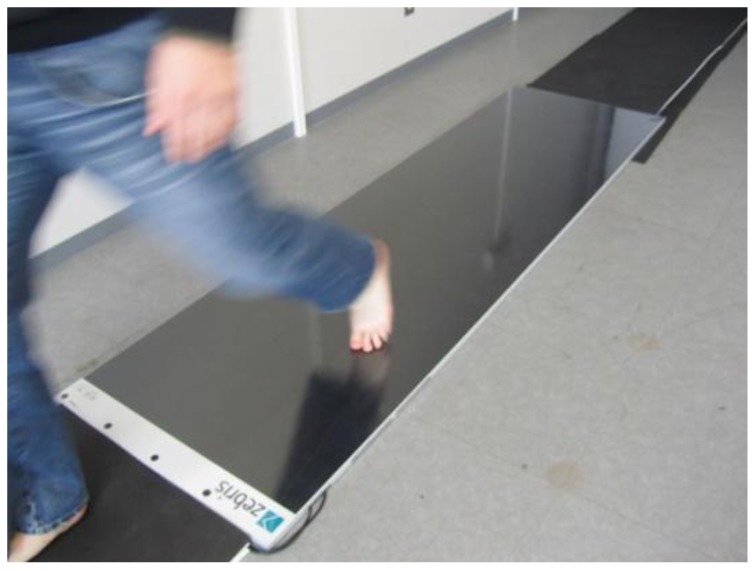
A platform based foot plantar pressure sensor by Zebris Medical GmbH [25].

Platform systems are constructed from a flat, rigid array of pressure sensing elements arranged in a matrix configuration and embedded in the floor to allow normal gait. Platform systems can be used for both static and dynamic studies but are generally restricted to research laboratories. One advantage is that a platform is easy to use because it is stationary and flat but has the disadvantage that the patient requires familiarization to ensure natural gait. Furthermore, it is important for the foot to contact the centre of the sensing area for an accurate reading [23]. Limitations include: space, indoor measurement, and patient's ability to make contact with the platform, Figures 1 and 2 show a platform-based sensor [24,25].

### 3.2. In-Shoe Systems

In-shoe sensors are flexible and embedded in the shoe such that measurements reflect the interface between the foot and the shoe. The system is flexible making it portable which allows a wider variety of studies with different gait tasks, footwear designs, and terrains [23].

**Figure 3.**
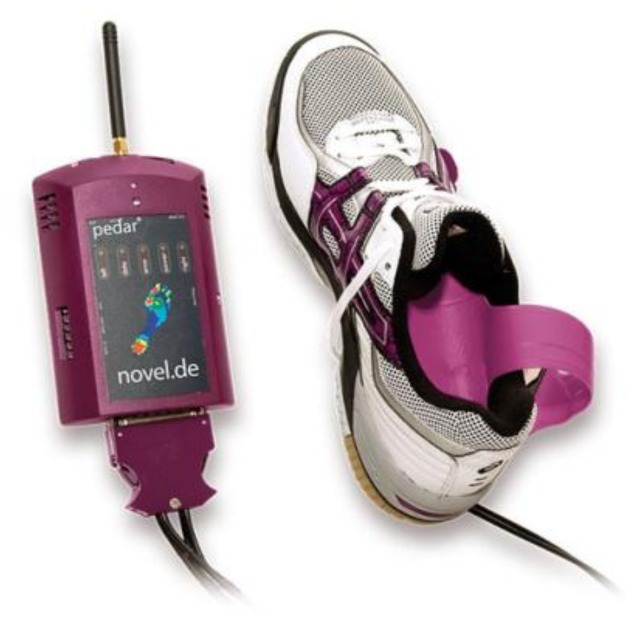
An in-shoe based foot plantar pressure sensor by Pedar^©^ Novel [24].

**Figure 4.**
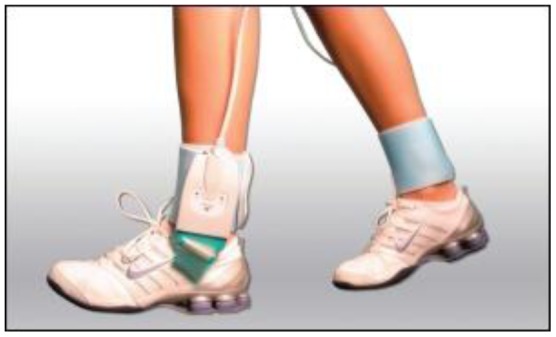
An in-shoe based foot plantar pressure sensor F-Scan^®^ System by Tekscan [26].

They are, therefore, highly recommended [11,23] for studying orthotics and footwear design but there is the possibility of the sensor slipping. Sensors should be suitably secured to prevent slippage and ensure reliable results. A further limitation is that the spatial resolution of the data is low compared to platform systems due to fewer sensors [11,23]. Figures 3 and 4 illustrate in-shoe based systems [24,26].

## 4. Requirement of Foot Plantar Sensors

In taking any biomechanical measurements, devices must be optimized for the specific application to ensure that readings are accurate. Detailed analysis must be thoroughly undertaken prior to any measurements and for foot plantar system two main considerations must be met; the target implementation requirements and the sensor requirements.

### 4.1. Target Implementation Requirements

**Figure 5.**
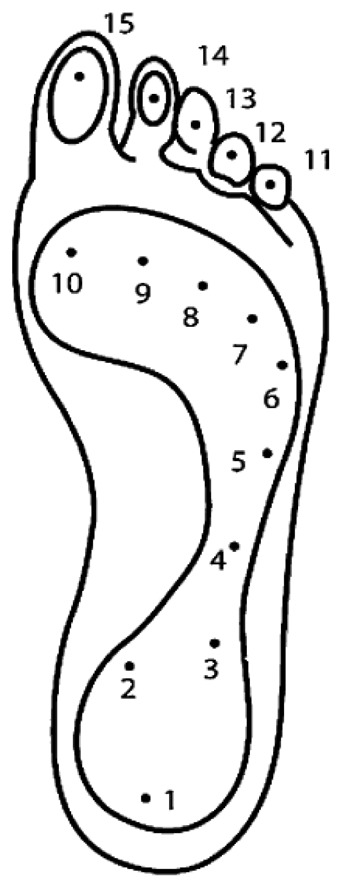
Foot anatomical areas [30].

Real-time measurement of natural gait parameters requires that sensors should be mobile, untethered, can be placed in the shoe sole, and can sample effectively in the target environment. The main requirements of such sensors are as follows:

1. *Very Mobile:* To make a sensor mobile, it must be light and of small overall size [27,28], the suggested shoe mounted device should be 300 g or less.
2. *Limited Cabling:* A foot plantar system should have limited wiring, wireless is ideal. This is to ensure comfortable, safe and natural gait [28].
3. *Shoe and Sensor Placement:* To be located in the shoe sole the sensor must be thin, flexible [29] and light [27]. It is reported that a shoe attachment of mass 300 g or less does not affect gait significantly [27]. Shu *et al.* [30] mentioned that the sole of foot can be divided into 15 areas: heel (area 1–3), midfoot (area 4–5), metatarsal (area 6–10), and toe (area 11–15), as illustrated in Figure 5. These areas support most of the body weight and are adjusted by the body's balance; therefore, ideally the 15 sensors are necessary to cover most of the body weight changes based on the Figure 5 anatomy.
4. *Low Cost*: The sensor must be affordable for general application [28] to benefit from inexpensive, mass-produced electronics components combined with novel sensor solutions.
5. *Low Power Consumption:* It should exhibit low power consumption such that energy from a small battery is sufficient for collecting and recording the required data.

### 4.2. Plantar Pressure Sensor Requirements

The key specifications for sensor performance include: linearity, hysteresis, sensing size, pressure range and temperature sensitivity [27,29,31]. Brief discussion of these is important as a basis for the selection of a sensor for specific applications.

**Figure 6.**
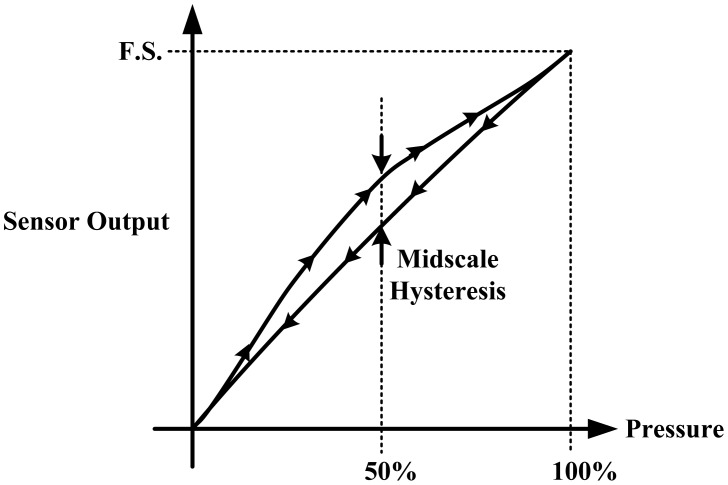
Hysteresis caused by loading and unloading a pressure sensor usually measured at the 50% pressure range. Adopted from [32].

**Figure 7.**
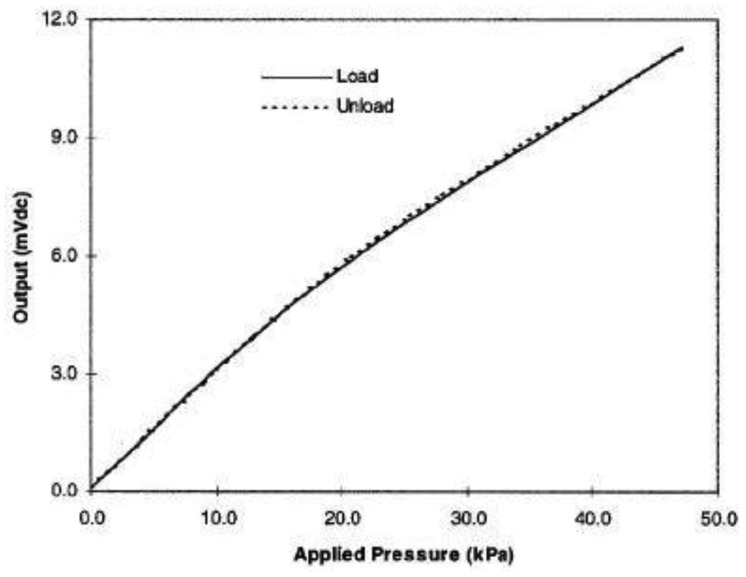
Negligible hysteresis of MEMS-based pressure sensor [29].

**Figure 8.**
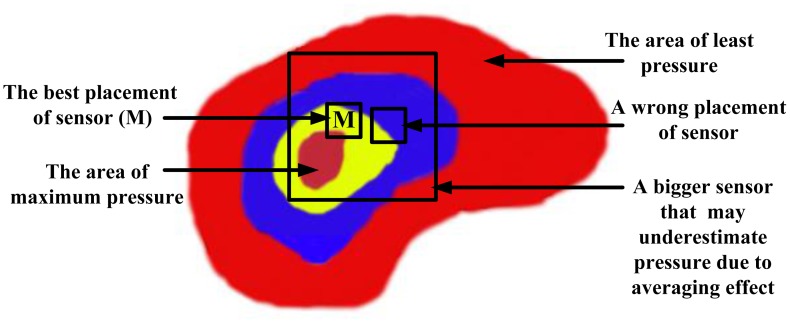
Effect of sensor sizing and placement.

**Figure 9.**
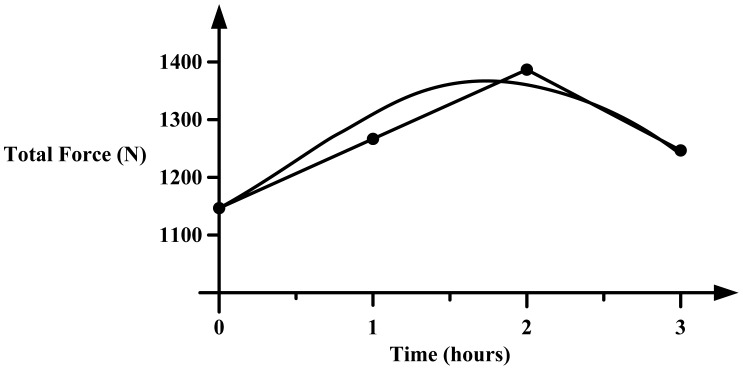
Example of erroneous readings due to sensor creep of Pedar^®^ Insole. The curve is the error reading by the sensor and plotted line is the correct pressure values. Modified from [35].

1. *Hysteresis:* Hysteresis can be determined by observing the output signal when the sensor is loaded and unloaded. When the applied pressure is increased by loading or decreased by unloading, two different responses are observed (Figures 6 and 7).
2. *Linearity:* The response of the sensor to the applied pressure, when plotted, will show the linearity figure of merit, *i.e.*, how straight the plotted line is. Linearity indicates how simple or complicated the signal processing circuitry will be, a highly linear response requires very simple signal processing circuitry and *vice versa*, a linear pressure sensor is, therefore, preferred.
3. *Temperature Sensitivity:* Sensors may produce different pressure readings as the ambient temperature changes. This may be due to the materials that are part of the sensor body as they respond differently to temperature change. A sensor with low temperature sensitivity in the 20 °C to 37 °C range is preferred [29].
4. *Pressure Range:* The pressure range is the key specification for a pressure sensor. As different applications require different operating pressures application-specific sensor development is normally adopted in the design. Maximum pressure is the upper limit that the pressure sensor can measure and *vice versa*. It is also important to note that burst pressure is the maximum pressure that the sensor can withstand before breakage as opposed to maximum pressure. Foot plantar pressure values of up to 1,900 kPa are typically reported in the literature but an extreme pressure of up to 3 MPa has been documented by Urry [31]. One of the foot plantar pressure sensor designs considers 3 MPa as burst pressure value, for comparison when a healthy person of 75 kg body mass is standing on only one forefoot, if pressure is evenly distributed, the interfacial pressure for every 31.2 mm^2^ foot plantar area approximates 2.3 MPa [33].
5. *Sensing Area of the Sensor:* Size and placement of the sensor are also critical, as shown in Figure 8. As a large sensor may underestimate the peak pressure and it is suggested that a minimum sensor of 5 mm × 5 mm should be used, whereas sensors smaller than this must be designed as array sensors.
6. *Operating Frequency:* It is recommended [31] that to measure foot plantar pressure precisely for running activities the sensors must be capable of sampling at 200 Hz. This frequency is generally considered sufficient for sampling most everyday gait activities.
7. *Creep and Repeatability:* Creep is the deformation of material under elevated temperature and static stress. It directly relates to the time dependent permanent deformation of materials when subjected to a constant load or stress [34], as in Figure 9. Low creep sensors are one of the key requirements in foot pressure measurement. Repeatability refers to the ability to produce reliable result even after long period of time [29]. High cyclic loads may cause deformation or fatigue [34]. Repeatability problems can be eliminated if the sensor exhibits no creep or deformation over repetitive or high cyclic loads.

## 5. Commercial Foot Plantar Pressure Measurement Sensors

There are several pressure sensors available on the market. Such sensor technologies utilize capacitive sensors, resistive sensors, piezoelectric sensors and piezoresistive sensors. These sensors provide electrical signal output (either voltage or current) that is proportional to the measured pressure. The required key specifications for a pressure sensor in terms of sensor performance include linearity, hysteresis, temperature sensitivity, sensing size and pressure range. The most common pressure sensors are capacitive sensors, resistive sensors, piezoelectric sensor and piezoresistive sensor.

### 5.1. Capacitive Sensors

**Figure 10.**
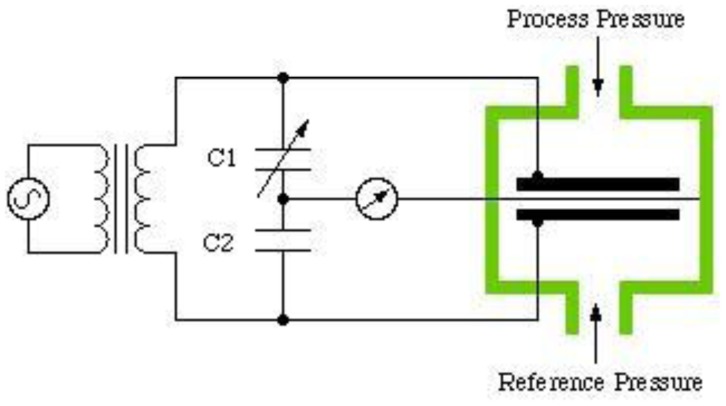
Capacitive pressure sensor construction [36].

The sensor consists of two conductive electrically charged plates separated by a dielectric elastic layer. Once a pressure is applied the dielectric elastic layer bends, which shortens the distance between the two plates resulting in a voltage change proportional to the applied pressure [11,31]. Figure 10 shows the capacitive sensor construction. Commercial products based on this system are the emed^®^ platform systems (Novel, Germany) [24] and Pedar^®^ in-shoe systems (Novel, Germany) [24].

### 5.2. Resistive Sensors

**Figure 11.**
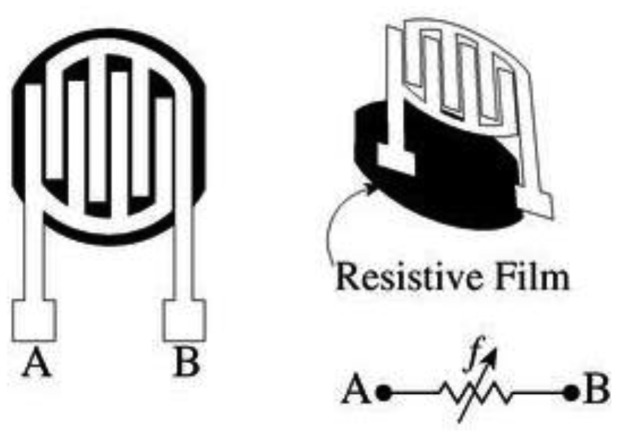
Resistive pressure sensor construction [37].

Force-Sensing Resistor (FSR) is a good example of the resistive sensor. When pressure is applied the sensor measures the resistance of conductive foam between two electrodes. The current through the resistive sensor increases as the conductive layer changes (*i.e.*, decreases resistance) under pressure. FSRs are made of a conductive polymer that changes resistance with force, applying force causes conductive particles to touch increasing the current through the sensors [11,31]. Figure 11 shows the resistive sensor construction and commercial products based on this principle are MatScan^®^ platform systems (Tekscan, USA) [26] and F-Scan^®^ in-shoe systems (Tekscan, USA) [26].

### 5.3. Piezoelectric Sensors

The sensor produces an electric field (voltage) in response to pressure. Piezoelectric devices have high impedance and therefore susceptible to excessive electrical interference that leads to an unacceptable signal-to-noise ratio.

**Figure 12.**
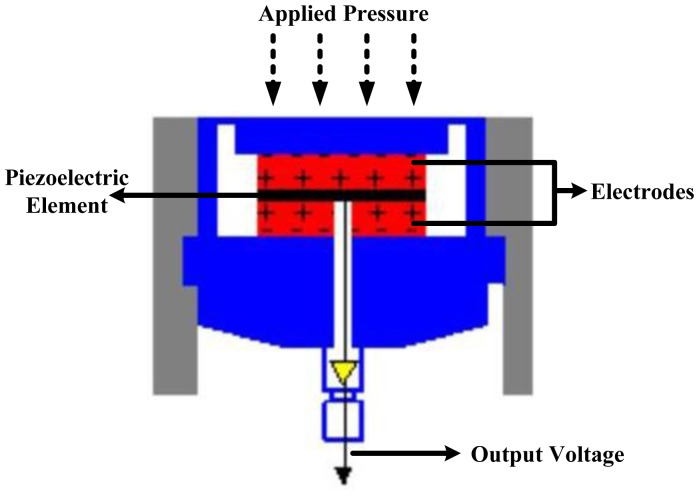
Piezoelectric pressure sensor construction [39].

The most suitable material for clinically oriented body pressure measurement is polyvinylidene fluoride (PVDF) because it is flexible, thin and deformable [11,31]. Figure 12 shows the piezoelectric sensor construction. Commercial products based on this system are Measurement Specialties, USA [38] and PCB Piezotronics, Inc., USA [39].

### 5.4. Piezoresistive Sensors

**Figure 13.**
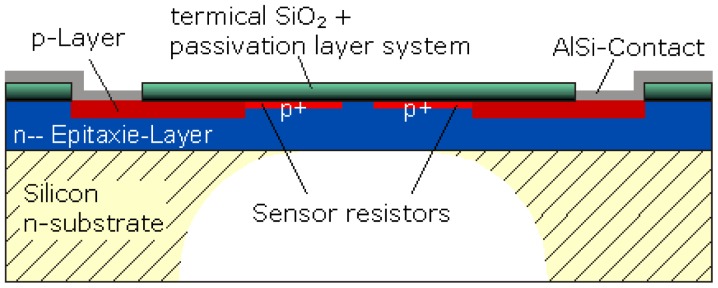
Piezoresistive pressure sensor construction [41].

This sensor is made of semiconductor material. In Piezoresistive material the bulk resistivity is influenced by the force or pressure applied, when the sensor is unloaded resistivity is high and when force is applied resistance decreases [11]. Figure 13 shows the piezoresistive sensor construction. When there is pressure on the piezoelectric element (quartz crystal) it produces electric charges from its surface. These charges create voltage proportional to the applied force. Commercial products based on this system are FlexiForce^®^ (Tekscan, USA) [26] and ParoTec (Paromed, Germany) [40].

The requirements of the pressure sensor for the specific application are low hysteresis, linearity of output, and pressure range [27,29,31]. A recommended pressure range for gait analysis (walking) is approximately 1,000 kPa [31] but for sports the pressure range should be larger due to the nature of the movements. There are a number of commercially available foot-pressure sensors on the market but they generally do not fulfil the requirements of many biomechanical applications due to specification and performance limitations. The limitations include but not limited to, the specified hysteresis [29], pressure span and physical sensor dimensions [42].

In comparison with traditional foot plantar pressure sensors such as capacitive sensors, resistive sensors, piezoelectric sensors and piezoresistive sensors the MEMS pressure sensors have many advantages. For example, easy communication with electrical elements in semiconductor chips, small size, lower power consumption, low cost, increased reliability and higher precision. To provide a better alternative developments of a specifically designed miniature foot pressure sensor based on MEMS technology have been explored. In response to the needs of such sensors, Wahab *et al.* [43] successfully designed, fabricated and tested a miniature foot pressure sensor based on MEMS technology that can be inserted in the insole of a shoe [43]. As reported by Wahab *et al.* [43] significant performance enhancements have been achieved, for example, the sensor is small, has high-pressure range measurement capability, and excellent linearity both at low and high pressures and possesses negligible hysteresis.

**Figure 14.**
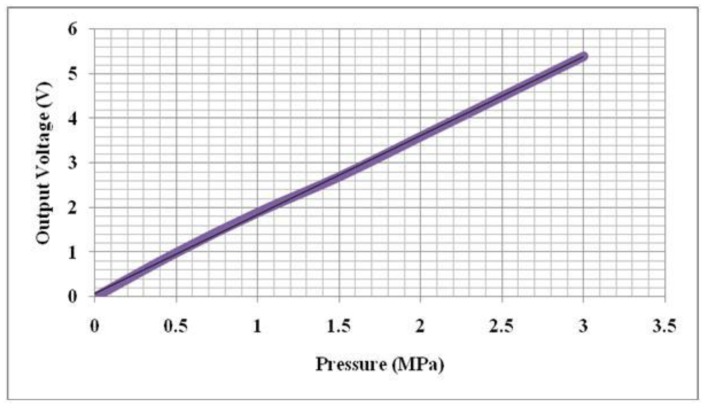
Graph demonstrating highly linear output voltage *vs.* pressure relationship.

**Figure 15.**
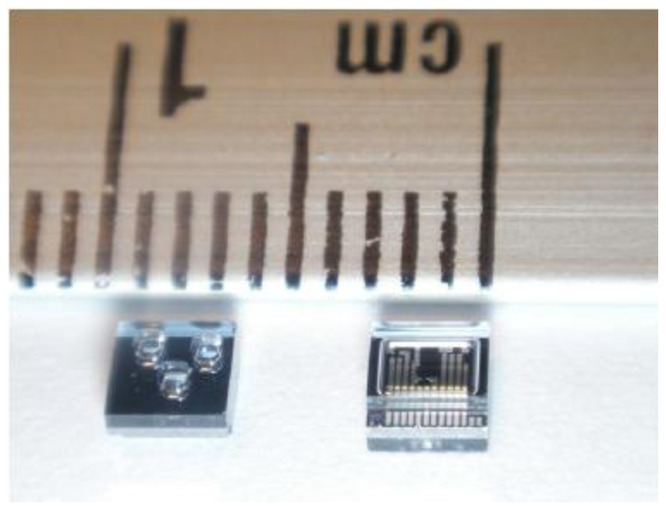
Picture of fabricated sensors.

Currently available in-shoe pressure sensor parameters are compared to Wahab *et al.* [43] in Table 1. Sensors from Vista Medical, Novel and Tekscan show some performance limitations as they are made of sheets of polymer or elastomer leading to issues such as repeatability, hysteresis, creep and non-linearity of the sensor output [29]. In addition to the above limitations some sensors (e.g., Parotec) have limited pressure range and relatively large dimensions. Figure 14 demonstrates the linearity of the MEMS based pressure sensor and the fabricated sensor as displayed in Figure 15.

## 6. Recent Trends in Foot Plantar Pressure Measurement

Trends in biomedical monitoring are toward using real-time and *in-situ* measurement of normal daily life parameters to keep pace with a fast-changing and demanding scientific environment. Gait analysis researchers are focusing on designing systems for uninterrupted measurement of real life parameters which is important in understanding the effect of daily activities on health. The ideal system to achieve this would be mobile, un-tethered, placed in the shoe sole and able to measure effectively in the targeted environment.

As early as the 1990s, Zhu *et al.* [46] developed a system for measuring the pressure distribution beneath the foot using seven force-sensitive resistors (FSR) and they used it to differentiate pressure between walking and shuffling [47]. In 1995, Hausdorff *et al.* [48] built a footswitch system capable of detecting temporal gait parameters using two FSR sensors. Later, in 1997, Cleveland Medical Devices Inc. [49] created an in-shoe wireless system which could measure time of foot contact, the weight on each foot and the centre of pressure (COP) of each foot. The system used a set of thick-film force sensors and since then there has been further development of in-shoe pressure sensor systems. In this paper, the focus is on the current development of the system.

### 6.1. Wired Systems Application

**Figure 16.**
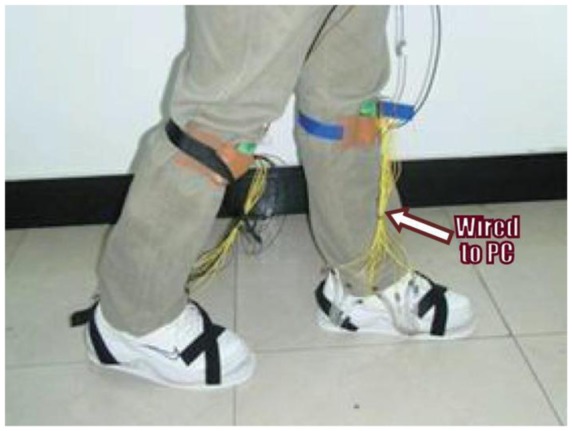
Identification based on dynamic plantar pressure in-shoe system [5].

Over the past two years there has been increasing interest in developing in-shoe foot plantar pressure systems and recently there have been applications to plantar pressure using both wired and wireless systems. In 2011, a paper employed dynamic plantar pressure for human identification using a FlexiForce^®^ (Tekscan, USA) in-sole pressure sensor [5]. They compared the pressure at different positions of key points then identified and classified them using a support vector machine (SVM) running on a PC. The system uses wire to transfer data from the sensor to a data acquisition card on a PC (Figure 16) and it is reported that the system has 96% identification accuracy.

**Figure 17.**
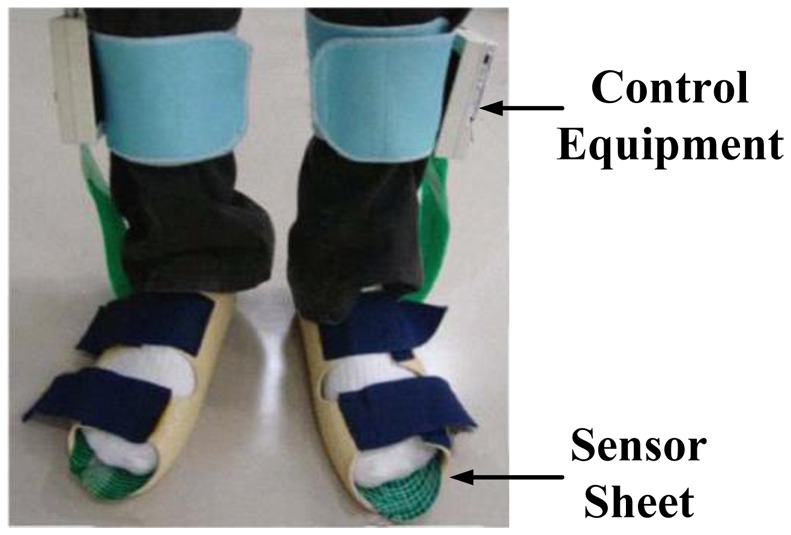
Biometric identification based on foot pressure pattern changes. Modified from [6].

Yamakawa *et al.* [6] also proposed their own biometric identification in-shoe system based on both feet pressure change and reported that the system could recognize over 90% of the test subjects. The system used F-scan (Nitta Corp, Japan) as the pressure sensor (see Figure 17).

Another innovative application is an in-shoe system to measure triaxial stress in high-heeled shoes [50]. The paper investigated the distribution of contact pressure and sheer stress simultaneously in high-heeled shoes utilizing five in-shoe triaxial force transducers commercialized by Anhui June Sport, China.

**Figure 18.**
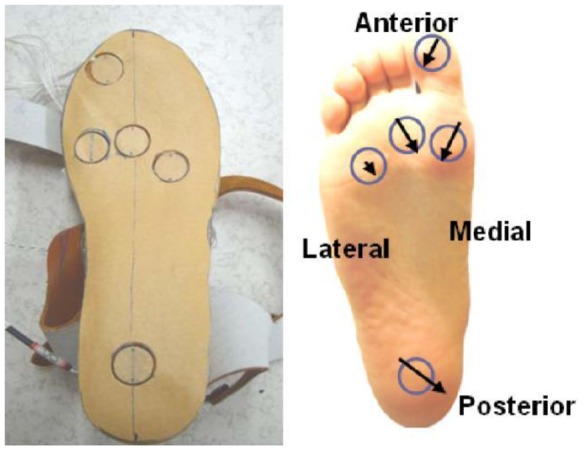
Mounted transducer in high-heeled shoe [50].

Shear stresses can cause blisters, callosities and trophic ulcers. The size of transducer is 17 mm × 18 mm × 10 mm and has 870 kPa full scale pressure range. In the system the transducer in mounted under the hallux, the first, second and fourth metatarsal heads as well as the heel as shown in Figure 18. As can be seen from the figure, peak sheer stress occurs at the second metatarsal; this type of information can be useful for future high-heeled shoe design.

Work undertaken by Healy *et al.* [51] claimed that their in-shoe system has a better repeatability compared to other commercially available systems. The sensor is practically similar to F-Scan^®^ (Tekscan, USA) which uses resistive force sensor.

**Figure 19.**
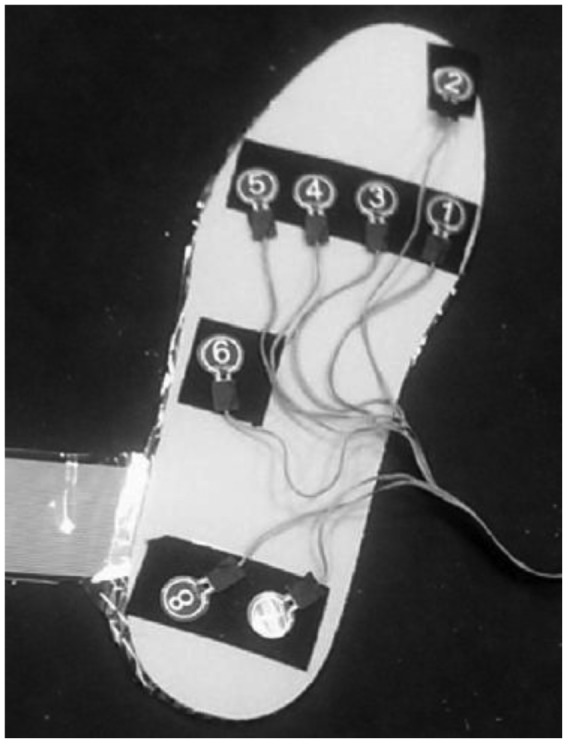
WalkinSense^®^ sensor placement [51].

**Figure 20.**
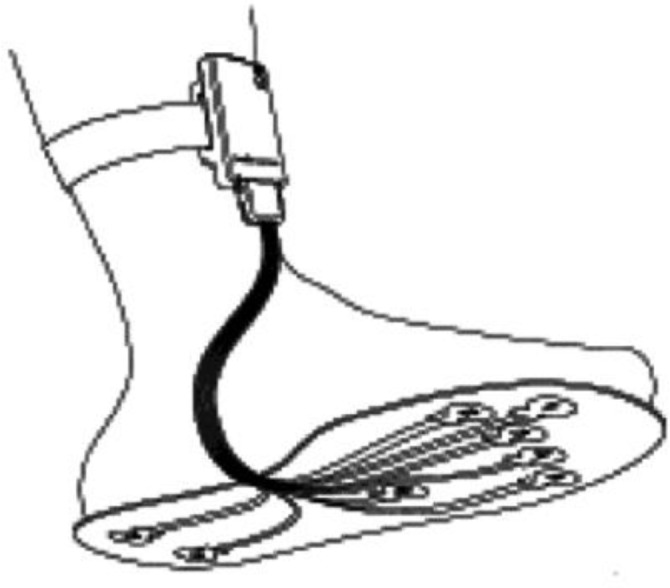
WalkinSense^®^ system [51].

The system is named “WalkinSense” and consists of a data acquisition and processing unit and eight individual sensors. It appears that only the sensor part is their own development, the rest of the system is similar to F-Scan^®^ (Tekscan, USA) hardware and software. The location of the sensors is illustrated in Figure 19, whilst the WalkinSense® System is shown in Figure 20.

All of the works described above [5,6,50,51] have a common feature that is wired to the processing unit or PC. All of them have certain benefits but for a wired system the major limitation is application in everyday monitoring. The wired system may encumber the test subject causing trip hazards or even a fall, and it can also affect the normal gait patterns. Therefore, it is recommended to make the system mobile for everyday usage, and the system must adapt to a wireless system. Research presented in [50,51] developed their own transducers but seems to have limited number of sensor placement. As mentioned in section IV, pressure recording from 15 locations is regarded as ideal for gait analysis. Papers [5,6] on the other hand used off-the-shelf sensors, which has also limitations as highlighted in Section 5.

### 6.2. General Wireless Systems Application

As mentioned earlier, there is a need for a system that can provide a wireless, real-time and reliable result to measure foot plantar pressure. There have been quite a few works undertaken in both research and commercial platforms that focused on developing a more mobile method of measuring foot pressure. Shoe based systems have been increasing, in terms of the number of publications and commercial products, due to shrinking in size of sensors, processing unit communication device and data storage. Further, the obvious reason for its usefulness is that such a system could measure the pressure distribution directly beneath the foot.

**Figure 21.**
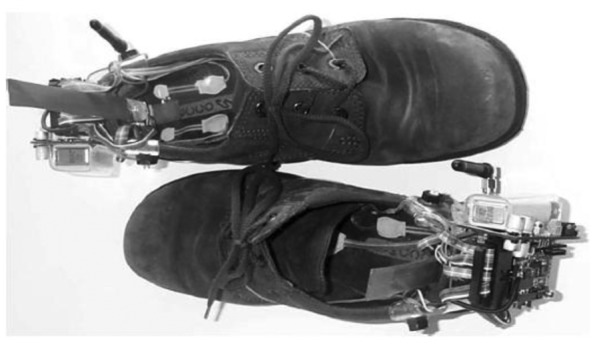
Shoe-integrated wireless sensor system, *GaitShoe* [27], showing all the hardware components.

The work undertaken by Bamberg *et al.* 2008 [27] from Massachusetts Institute of Technology (MIT), had been mentioned in a large number of the papers that were reviewed. The main reason why Bamberg *et al.* have received so much attention in the literature is that they had come up with arguably the most complete wireless in-shoe system for gait analysis to date. They called it *GaitShoe*. In their system, the sensors included three orthogonal accelerometers, three orthogonal gyroscopes, four force sensors, two bidirectional bend sensors, two dynamic pressure sensors and electric field height sensors. The devices were capable of detecting heel-strike, estimating foot orientation and position and toe-off. The microcontroller (Silicon Laboratories), RF Monolithic (as the transceiver), antenna and power supply were also attached to the shoes. Figure 21 displays the *GaitShoe* with all the hardware mounted on the shoes.

**Figure 22.**
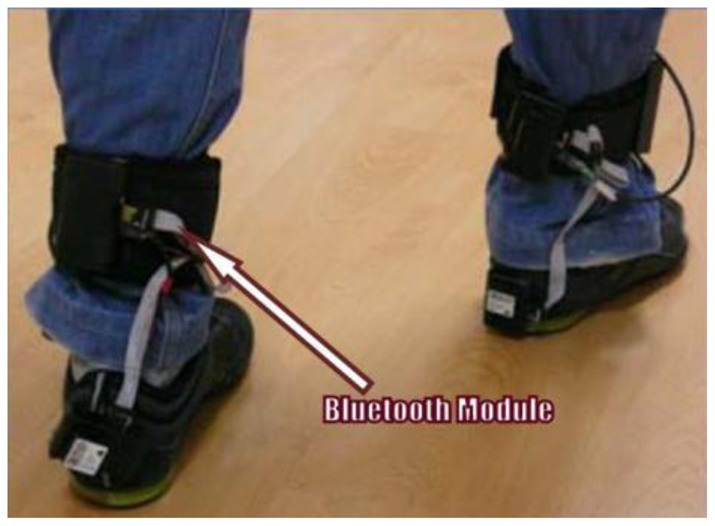
A wireless systems for gait and posture analysis based on pressure insoles and inertial measurement units [52].

In 2009, Benocci *et al.* [52] from University of Bologna, Italy developed a wireless system for gait and posture analysis. The wearable system utilised 24 hydrocells (by Paromed) to measure the plantar pressure and inertial measurement unit (IMU) in each shoe insole. The IMU integrated a 3-axes accelerometer and a digital 3-axes gyroscope. To control the system, Texas Instrument MPS430 microcontroller was implemented and Bluetooth acted as the transceiver. The collected data from the sensor allowed the user to recognize walking phases such as swing and stance, step and stride duration, double support and single duration. Figure 22 shows Benocci *et al.* wireless gait shoe.

**Figure 23.**
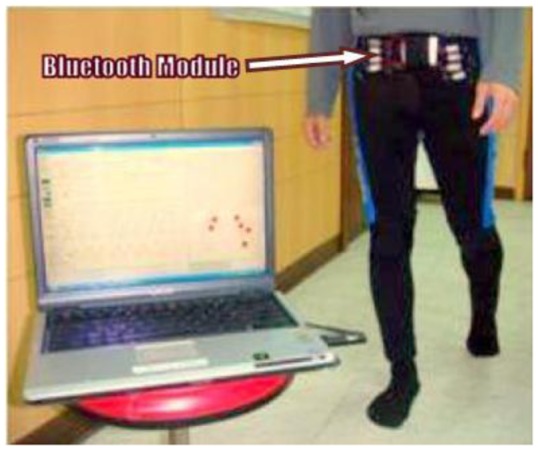
A wireless gait analysis system by digital textile sensors [53].

Ming Young Biomedical Corp., Taiwan published a state-of-the-art digital textile sensor for measuring gait analysis [53]. Four dome shaped sensors were knitted on each sock. The dome shape sensors were able to record spatio-temporal plantar pressure patterns which were used to calculate the centre of pressure (COP) excursions. Five clip type sensors were sewn to the pant to record lower limb movement. The system was reported to measure the duration of stride cycles and left/right steps, cadence, walking speed, and COP. The microcontroller (Texas Instrument MPS430) and Bluetooth were attached to the wearer's belt. Figure 23 portrays the digital textile sensors in action.

Shu *et al.* [30] developed an in-shoe plantar pressure measurement and analysis system based on fabric pressure sensing array in collaboration with Hong Kong Research Institute of Textiles and Apparel Ltd. The sensors used were textile fabric sensor array, which is soft, light and has high pressure sensitivity. The sensors were connected with a soft polymeric board through conductive yarns and integrated into the insole.

**Figure 24.**
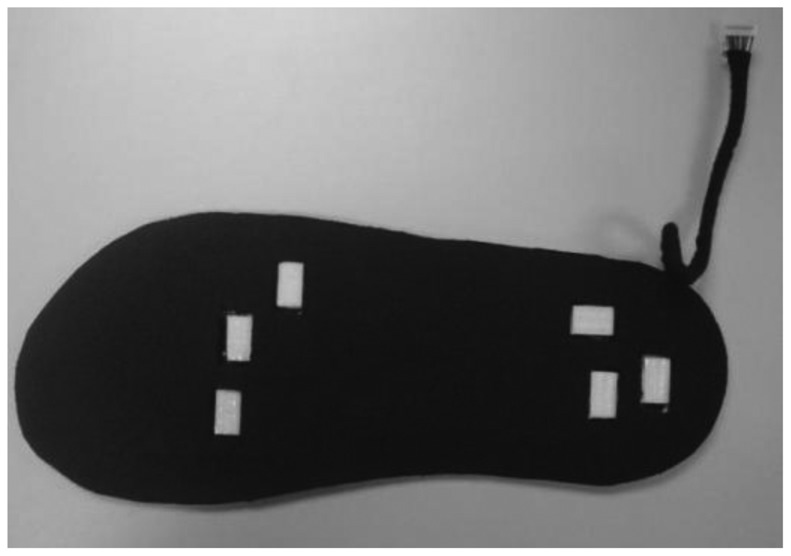
Fabric pressure sensing array indicating sensor placement [30].

**Figure 25.**
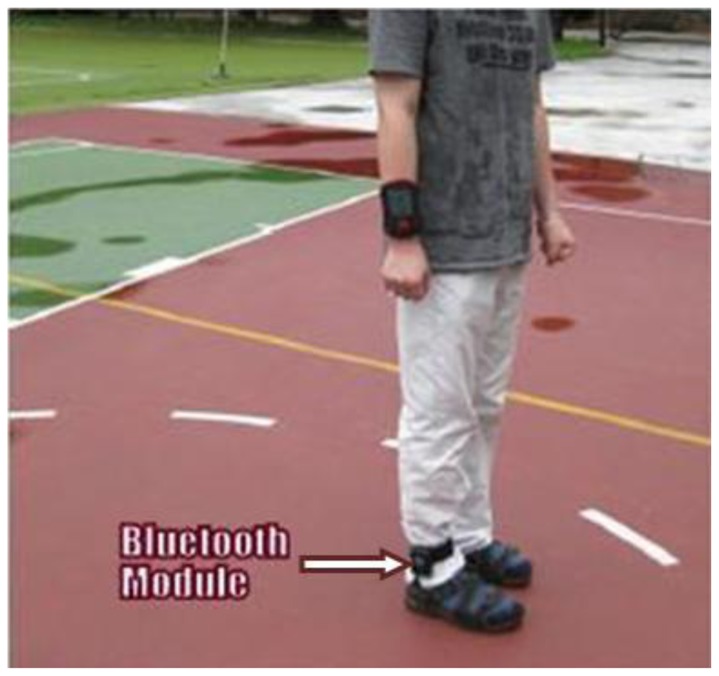
In-shoe plantar pressure measurement and analysis system based on fabric pressure sensing array [30].

Sensors were attached to six locations in the insole, as shown in Figure 24. The microcontroller PIC18F452 and the Bluetooth module were attached to the ankle of the patient. The system could interface with desktop, laptop and smart phone and was able to calculate parameters such as mean pressure, peak pressure, COP and shift speed of COP. The results were presented for both static and dynamic measurement conditions. Figure 25 shows the in-shoe plantar pressure measurement and analysis system based on fabric pressure sensing array.

The developments of wearable wireless sensor system for measuring foot plantar pressure have been encouraging. There is no doubt about their application potentials, especially the biomechanics communities. Nearly all use off-the-shelf sensors, microprocessors and wireless transmitters, so the end product is bulky and not comfortable to wear by the patients. The digital textile sensors by Chang-Ming *et al.* [53] are small and really flexible but it is wired to a Bluetooth based transmitter device at the belt. Both Benocci *et al.* [52] and Lin Shu *et al.* [30] used Bluetooth modules to attach to the ankle. Even more uncomfortable would be the system proposed by Bamberg *et al.* [27] where the whole sensor and wireless communication tools are attached to the top of the shoe. Although Bamberg *et al.* has developed the in-shoe gait analysis system but the system is not wearable for daily activities monitoring.

### 6.3. Major Application Areas

#### 6.3.1. Rehabilitation Applications

**Figure 26.**
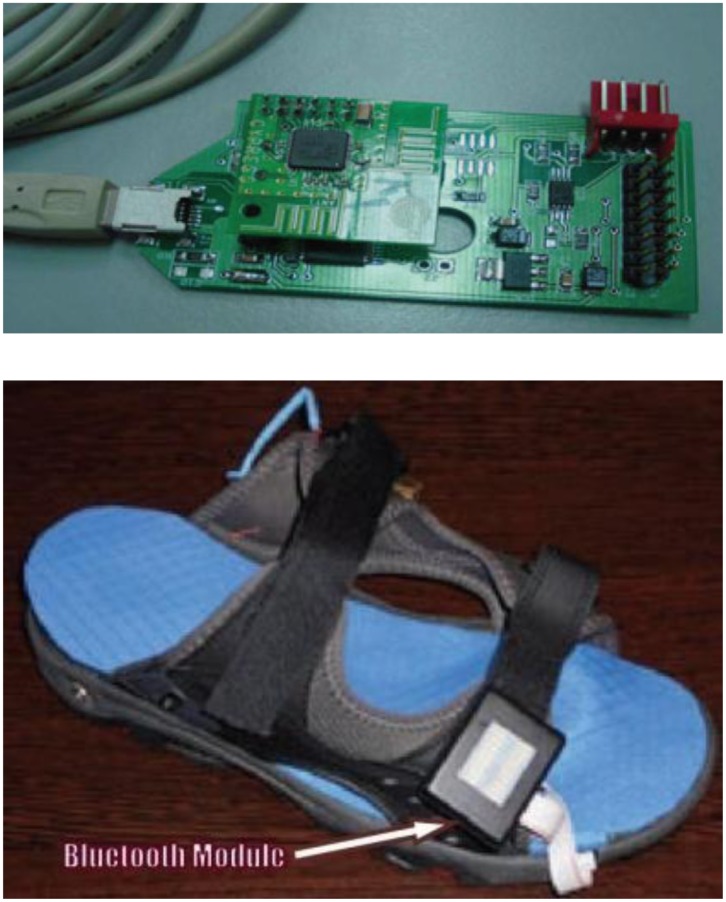
Neaga *et al.* [8] microcontroller board and shoe prototype.

**Figure 27.**
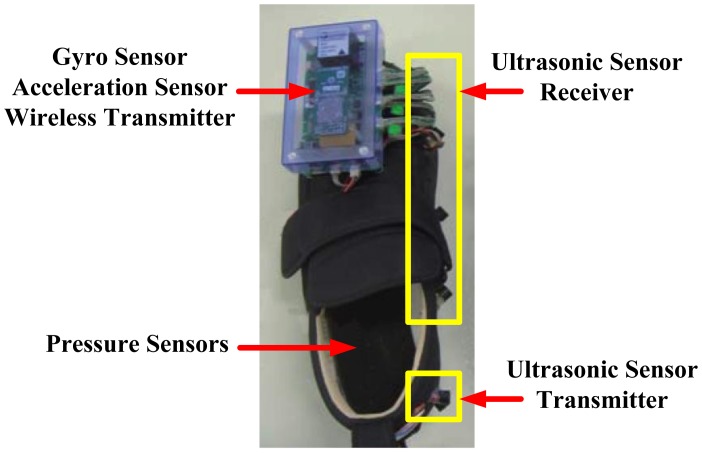
“GaitGuide” shoe prototype. Modified from [9].

**Figure 28.**
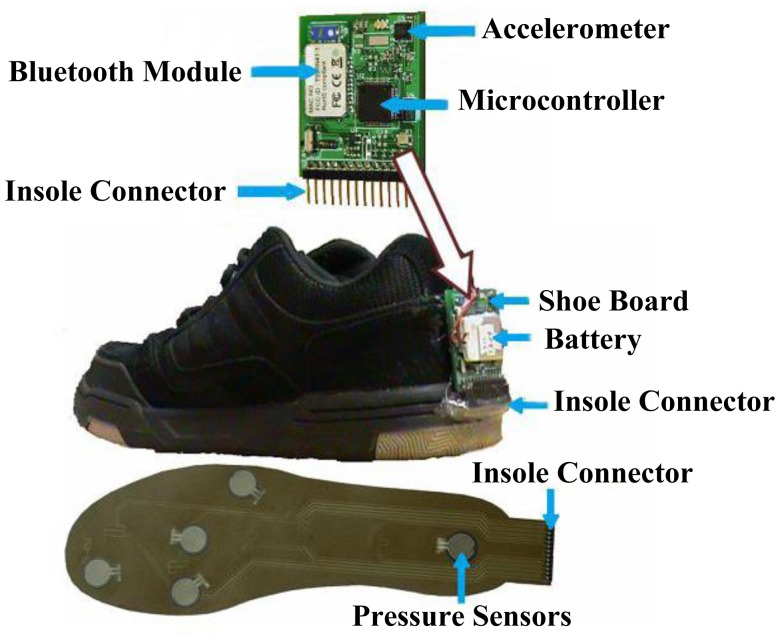
Edgar *et al.* [10] shoe prototype.

Wireless foot plantar systems have been applied to a number of areas including rehabilitations, sports and daily life gait monitoring. For example, Crosbie and Nicol [54] indicated that as part of rehabilitation procedure of patients with spinal cord injury and diabetes, it is quite useful to measure the efforts exerted by lower limbs such as plantar surface pressure/force distributions and the contact sensation with the ground. This information is essential in providing better rehabilitation strategies. Recent publications on wireless systems for rehabilitation applications include work by Neaga *et al.* [8] for monitoring the progressive loading of lower limb in post-traumatic rehabilitation, by Wada *et al.* [9] for rehabilitation support system and by Edgar *et al.* [10] on wearable shoe for rehabilitation of stroke patients. Neaga *et al.* used F-Scan^®^ (Tekscan, USA) for the sensor, microcontroller based data acquisition and RF transceiver for wireless communication. The system indicates to the user excessive loading of the lower limb through LED indicators. Figure 26 presents the prototype shoe and the prototype hub. Wada *et al.* developed their system named “GaitGuide”. The system used sensors units (gyro sensor, acceleration sensor, ultrasonic sensor and pressure sensor), a wireless module, an electronic tag to collect data from the shoe and display gait information. The “GaitGuide” could collect the gait information in the form of step length, step width and pressure. The gait information can be used to design a specific rehabilitation program for a particular patient. The “GaitGuide” prototype is displayed in Figure 27. Edgar *et al.* [10] indicated that their system can classify patient's recovery from stroke posture. They claim that the system had 99% accuracy in the classification. The system applied a microcontroller (Texas Instrument, USA), a Bluetooth module (Roving Network, USA), accelerometer sensor and in-sole pressure sensor. Figure 28 shows components of the developed prototype.

These published researches [8–10] are all wireless, and all are designed to assist patients with mobility problems. Figures 26–28, however, indicate that these bulky electronics may not be suitable for monitoring recovery in post-traumatic patients. Another point worth noting is that these three systems used commercial foot plantar pressure sensors, and the limitations of commercial sensors have been highlighted in Section 5.

### 6.4. Sport Applications

**Figure 29.**
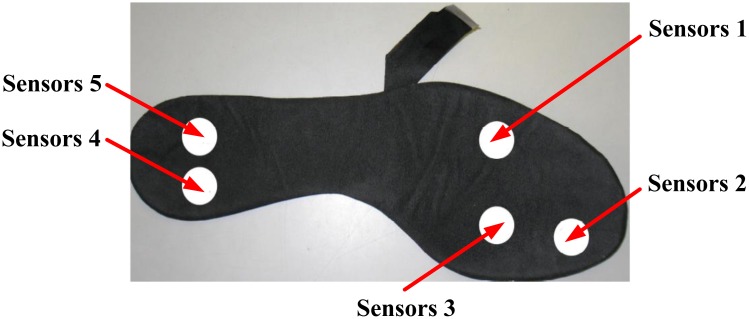
The five sensors placement of Salpavaara *et al.* designed system. Modified from [55].

**Figure 30.**
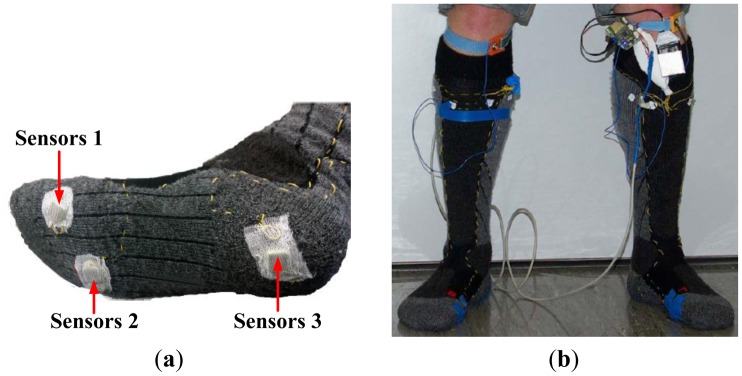
The three sensors placement of Holleczek *et al.* sensor sock designed. (**a**) three sensors placement; and (**b**) wearable system. Modified from [56].

Another application that relies on a wireless system is sport application. Noteworthy mentions are research by Salpavaara *et al.* [55] and Holleczek *et al.* [56]. These two papers employed innovative new sensors for their application using custom made laminated capacitive sensor matrix and textile pressure sensors respectively. Salpavaara *et al.* system can be utilized to monitor the timing and movement of the legs of the athlete during throwing, jumping and running in various sports events. The obtained data can be used for improving sports coaching. They opt for javelin throwing for their case study. In their case study they conclude that the timing of the steps, support and release phase has a great importance in the performance and the pace of steps should increase towards the end of the throw event. In their system they employ a capacitance-to-digital converter (Analog Device, USA), microcontroller (Atmel, USA) and a Zigbee-compliant radio. Figure 29 shows the five sensors placement. Holleczek *et al.* developed “SnowPro”, a wearable sport trainer, capable of supporting snowboarders in improving their skill. The system is able to analyze the dynamics of the weight distribution inside the boots. This type of information is essential for identifying the wrong weight shifting techniques, which usually lead to painful crashes in snowboarding sport. The system gives feedback to the user in real-time or after the activity about user performance and support user during learning process. This system utilizes three integrated textile pressure sensors, six capacitance-to-digital converters and a Bluetooth module. Figure 30 displays the final design by Holleczek *et al.* [56].

The obvious deficiency in the system is the number of sensors used. Salpavaara *et al.* [55] used five and Holleczek *et al.* [56] used only three. For many sport biomechanics applications this number may not be adequate. Besides that, for sport applications the system should not obstruct the athlete's movement. From Figure 30(b) it is apparent that it is not very practical. This is due to the fact that both systems utilized off-the-shelf equipment that is usually very bulky.

#### 6.4.1. Other Wireless Systems Application

**Figure 31.**
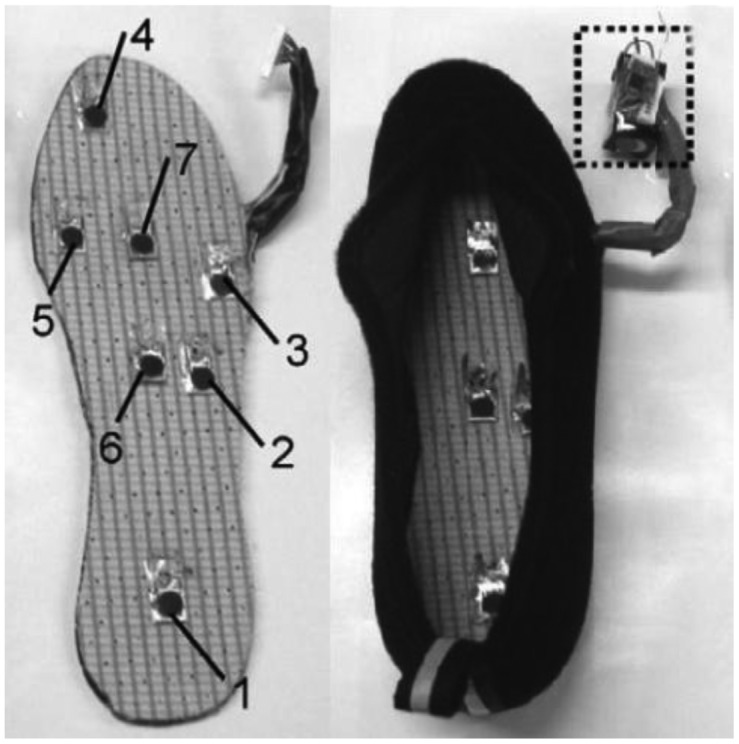
The seven sensors placement and the complete shoe. The box inlet is the power source, wireless transmitter and pressure measurement unit [57].

Other wireless in-shoe foot plantar pressure system that can be highlighted are those proposed by Saito *et al.* [57] and De Rossi *et al.* [58] which employ unique pressure sensors to measure plantar pressure during daily human activity. Saito *et al.* device consists of a shoe insole with seven pressure-sensitive conductive rubber (PSCR) sensors (Yokohama Image System, Japan), 10-bit analog-to-digital converter and a RF wireless transmission unit. Each (PSCR) sensor is about 15 mm × 10 mm × 0.8 mm, and can measure pressure in the range 25–250 kPa. The seven sensors are placed at heel, lateral midfoot, great toe, head of the first metatarsal, centre midfoot and centre forefoot as portrayed in Figure 31. Figure 31 also displays the complete shoe with the power source, wireless transmitter and pressure measurement unit.

Saito *et al.* [57] system has several benefit over the other system, namely they didn't adopt a processing/microcontroller unit attached to the shoe ensuring the electronic circuitry is kept small, and their processing unit is at the receiving end of the system. This benefit also makes the power consumption lower compared to other systems, thus the system is capable of monitoring up to 20 hours with changing power source. On the contrary, the sensor has limited pressure range; the maximum the transducer could sense is 250 kPa. A typical obese person can generate more than 500 kPa average peak pressure for both men and women [59] and for sport application for instance during triple jump it is reported that the maximum pressure can reach around 750 kPa to 1 MPa depends on the athletes [60].

**Figure 32.**
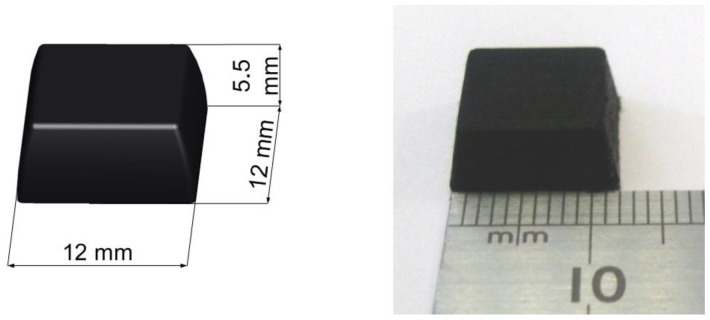
De Rossi *et al.* sensor dimension.

**Figure 33.**
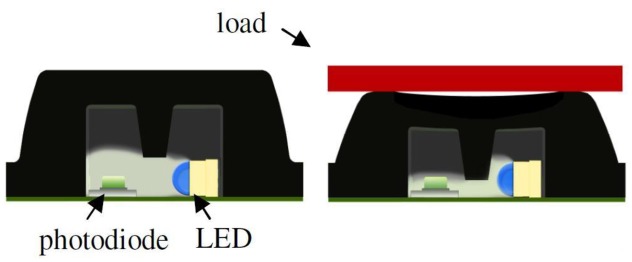
Working principle of De Rossi *et al.* sensor.

**Figure 34.**
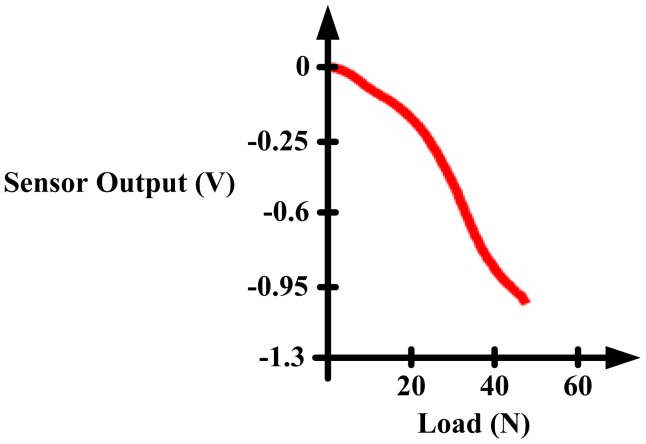
De Rossi *et al.* sensor characterization: Force *vs.* Output Voltage.

**Figure 35.**
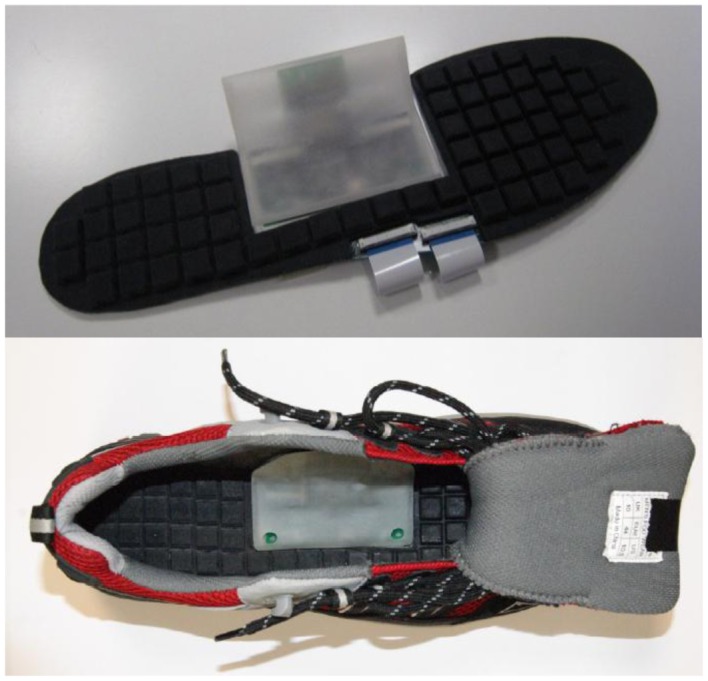
De Rossi *et al.* insole sensor system and the system fitted inside a shoe.

De Rossi *et al.* [58] employed 64 silicone-covered opto-electronic pressure sensors array, four 16-channel 14-bit analog-to-digital converters, a microcontroller and a Bluetooth module. The sensors have 12 mm × 12 mm × 5.5 mm dimension, maximum loading of 500 kPa without damaging the sensor. Figure 32 shows the dimensions of the sensor. The transduction principle of the sensors is demonstrated in Figure 33. When a load is applied to the top face, the cover causes a deformation, and lowers silicone ‘curtain’ which obstructs the light path from the LED to the photodiode. Therefore, lower the light from the LED receiving at the photodiode producing lower voltage at the output of the photodiode. Thus, it is inversely proportional the relationship between input force and output voltage. The sensors also show no significant static hysteresis. Figure 34 plots the force *vs.* output voltage characterization. Figure 35 depicts the complete insole pressure system and the system fitted inside a shoe.

Based on the information in reference [58] the sensor design by De Rossi *et al.* has a number of advantages. The advantages are the number of sensor placement nearly covers the whole surface of the foot, the bulky electronics board is well hidden in the medial arch of the foot and the sensor has no significant hysteresis. On the flipside, the sensors has a bad linearity at low and high pressure which will require a more complex signal processing to ascertain a more accurate representation of the pressure. Another downside is that the sensor has limited life expectancy, the sensor will damage if a pressure exceeding 1 MPa is forced on it. The housing for the electronic board is made out of thin PCB, which the authors mention that it is comfortable to wear.

From the review there is one common limitation in most of these systems, which is the wireless transmitter/transceiver. If only the wireless transmitter/transceiver could be integrated and minimized the size it could be inserted inside the insole of the shoe with the entire sensor. This would make the shoe more wearable for daily life activities, and help with diagnosing foot problems.

## 7. Proposed Wireless DAQ Foot Plantar Pressure System

**Figure 36.**
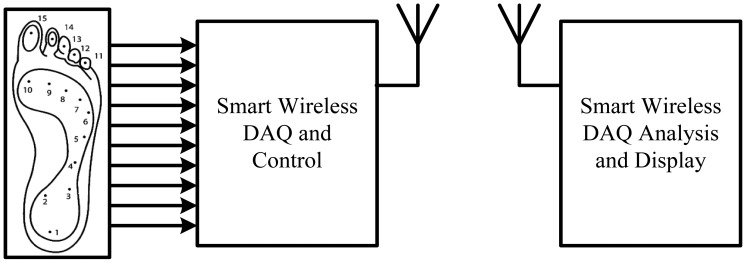
Block diagram of the proposed system.

Based on the reviewed current in-shoe foot plantar systems, it seems there are some limitations that could be improved. One area of the improvement could be development in the wireless data acquisition (DAQ) for the in-shoe foot plantar pressure sensor system. So we propose to design and implement miniaturised insole, low power and wearable wireless system using customized MEMS sensors for measuring foot plantar pressure and interface it with wireless DAQ unit that can be also slotted in the in-sole of the shoe. The research work requires a systematic understanding of different types of wireless systems on chip, the requirement of MEMS pressure sensor for measuring foot plantar pressure and realistic scenarios for their implementation and the application. The MEMS pressure sensors have several advantages compared to others such as small in size, high pressure range, linear and high reliability. The specific aim of the research is to design a wireless foot plantar pressure measurement system. The transmitter must be compatible with the MEMS sensor, meet the requirement of measuring foot plantar pressure analysis and wearable for in-shoe applications. The receiver should be suitable for interfacing with data logger, desk-top or lap-top for further data analysis. Figure 36 shows the block diagram of the proposed system.

**Figure 37.**
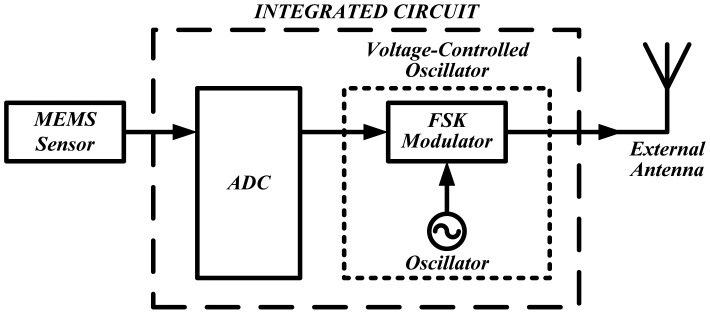
Block diagram of system design.

**Figure 38.**
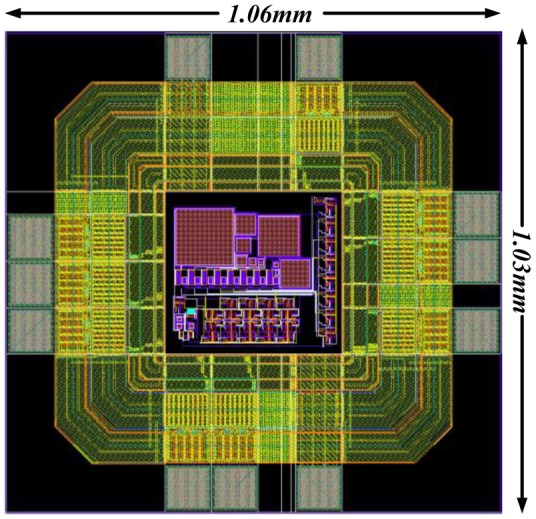
The layout design with padding.

A wireless DAQ-IC which can be inserted in the insole of a shoe has been designed and simulated [61]. The system architecture is shown in Figure 37. In this design the first task was to transmit data from only a single MEMS sensor. The layout design of the IC is displayed in Figure 38. The total chip size of the design including pads is about 1 mm^2^.

**Figure 39.**
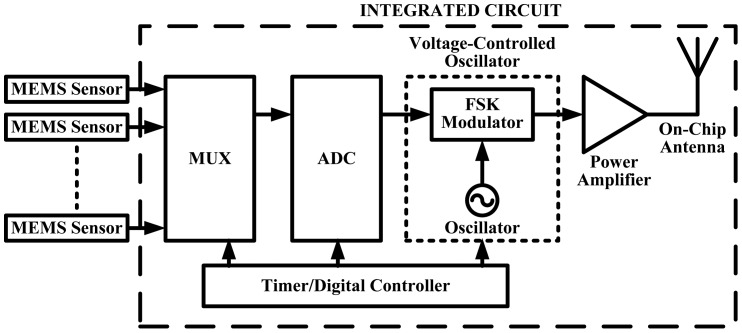
The new proposed system design block diagram.

For further improvement of the system, we added more features based on our earlier design. First, including an analog multiplexer (MUX) to ensure the single DAC chip can cater for all 15 sensors based on our initial block diagram. Second, after the inclusion of the MUX a controlling unit is added to the design (Figure 39) to control the whole system design making it a smart system and finally, we integrated an on-chip antenna thus creating the whole wireless DAQ system in a single chip with only the addition of the power supply. The new proposed design is depicted in Figure 39.

## 8. Conclusions and Future Work

This paper has reviewed major foot plantar pressure measurement systems reported in the current literature and available in the market. Firstly, it discussed the available plantar pressure sensors. Then, it reviewed the latest research on wearable hardwired and wireless sensor systems for gait analysis and discussed their limitations. Finally, it presented a proposed solution for wearable wireless sensor systems for sensing foot plantar pressure. Initial IC design results show potential for some good results by the proposed in-shoe foot plantar pressure measurement system. A power consumption of 19.53 mW was achieved using charge redistribution successive approximation architecture as the DAQ and a ring VCO as the FSK modulator. The total chip size of the design including pads is about 1 mm^2^. Analysis of the full sensor node power consumption showed that this wireless DAQ is sufficient for the intended system operations. These experimental results are encouraging and show that the system is feasible for converting the sensor data to digital signals, and hence translating it to its frequency representation and ready for transmitting. Although the design managed to minimise the dimension, among other significant considerations is power consumption. Whilst 20 mW is moderately low, in today's microelectronics it is still pretty high. Therefore, the system will be considered to redesign focusing on the power consumption with a smaller CMOS design process before submitting to a fabrication foundry. The change in process might also create a domino effect in terms of the output parameter so the new design might need to add more features on the system such as including a power amplifier for increasing the signal power before transmitting and redesign the VCO to meet the target ISM frequency band for transmission purposes.

## Figures and Tables

**Table 1.** Commercially available in-shoe pressure sensors compared to Wahab *et al.* sensor.

	**Vista Medical** [44]	**Tekscan** [26]	**Novel** [24]	**Parotec** [45]	**Textile Sensor** [30]	**Wahab *et al.*** [43]
**Sensor Size**	2 mm thick	0.15 mm thick	1.9 mm thick	∼4 cm^2^ (hydrocell)	Not Specified	2 mm thick
**Number of Sensor**	128 (in shoe)	960 (insole)	99 (insole)	24 (insole)	6 (insole)	15 (insole)
**Range (kPa)**	260	1,034	1,200	625	800	3,000
**Frequency (Hz)**	Not Specified	500	Not Specified	250	100	200
**Hysteresis**	Not Specified	24%	<7%	0.05% at 20 Ncm^−2^	Not Specified	Negligible
